# Nutrient Analysis of Raw United States Beef Offal Items

**DOI:** 10.3390/nu16183104

**Published:** 2024-09-14

**Authors:** Hannah F. Fuerniss, Cody L. Gifford, Emma G. Mortensen, Keith E. Belk, Terry E. Engle, Dale R. Woerner

**Affiliations:** 1 Center for Meat Safety & Quality, Department of Animal Sciences, Colorado State University, Fort Collins, CO 80523, USA; keith.belk@colostate.edu (K.E.B.); terry.engle@colostate.edu (T.E.E.); 2 Department of Animal Science, University of Wyoming, Laramie, WY 82071, USA; cody.gifford@uwyo.edu; 3 Department of Animal & Food Sciences, Texas Tech University, Lubbock, TX 79409, USA; emmamortensen.rdn@gmail.com (E.G.M.); dale.woerner@ttu.edu (D.R.W.)

**Keywords:** beef, offal, variety meat, nutrient, analysis, nutrition

## Abstract

Nutrient composition of beef offal was evaluated to expand availability of nutrient data for the following beef items: beef heart, liver, kidney, tongue, honeycomb tripe, oxtail, marrow bones, testicles, and blood. These items are consumed both domestically and internationally, with significant regional variations that can be contributed to unique cultural meals and dietary patterns. Standardized procedures were used to dissect and homogenize beef offal samples. Nutrient analysis occurred at United States Department of Agriculture Agricultural Research Service-approved laboratories using validated methods and standards. Each of the offal items in the study qualified for at least one “Good Source” or “Excellent Source” nutrient labeling claim as defined by the United States Food and Drug Administration, based on composition of the separable lean component. This study provides analytically derived nutrient information for U.S. beef offal items. The results reflect that these products could be beneficial in providing essential nutrients as a component of a healthy diet.

## 1. Introduction

Consumers’ dietary decisions are multifaceted, from seeking enjoyment, convenience and affordability, to considering potential health implications. Across generations, many people desire health benefits from the foods they choose to consume, including decreased fatigue, weight loss, and improved cognition [1]. The 2020–2025 Dietary Guidelines for Americans placed emphasis on the idea of long-term dietary choices for sustained health and quality of life by focusing on healthy eating patterns across the lifespan, rather than on specific foods or nutrients as earlier guidelines had focused on. A major message of recent editions of the Dietary Guidelines is to enjoy a variety of nutrient-dense foods across all food groups to aid in meeting nutrient needs while also customizing dietary choices to reflect budgetary constraints, preferences, and traditions [2,3]. Beef offal items contain essential nutrients, including protein as well as various vitamins and minerals, and are also customary in traditional cuisine of various cultures [4,5,6].

In order to make informed nutrition-related decisions and recommendations, from the individual to the policy level, it is essential to have an accurate source of nutrient data for all foods. Partnerships between industry groups, academic institutions, and federal agencies have allowed for the expansion and improvement of nutrient composition data for the benefit of numerous stakeholders. The National Cattlemen’s Beef Association, a contractor to the Beef Checkoff, has previously supported compilation of nutrition information for beef muscle cuts [7]. However, prior to this study, current nutrition information was not available for beef offal items, which can be described as edible parts of the carcass other than skeletal muscle and are also known as variety meats, primarily in the United States.

Consumer attitudes regarding offal vary widely, with familiarity and background playing key roles [8,9]. Nonetheless, beef offal is consumed around the world with certain regions leading consumption of specific products based on cultural meals and traditions. Consumers in Mexico and the Southwestern United States regularly cook with tripe, while oxtail is more commonplace in the Northeastern US [4]. The majority of beef tongue and liver produced in the U.S. are exported overseas, garnering premiums compared to the domestic market for these products [4]. Japan is the leading destination for beef tongue, and liver is heavily consumed in Egypt in addition to regions of Africa as well as Latin America [4]. These products may be of significant value for nutrition security of the growing global population [5,10].

Regardless of the population ultimately consuming these beef offal items, accurate nutrient data is essential to better understand their contribution to the diet and to inform nutrition recommendations. However, information on the nutritional profile of edible offal was recently described as “scattered and limited” [5]. Therefore, the objective of this study was to expand the availability of nutrient composition data for U.S. beef offal.

## 2. Materials and Methods

Institutional Animal Care and Use Committee approval was not required for this study, as samples were obtained from federally inspected harvest facilities. Dissection and analysis procedures used for this study were nearly identical to methods described by Acheson et al. (2015) [11] and Gifford (2016) [12].

### 2.1. Sample Collection and Preparation

#### 2.1.1. Product Collection

Product sampling was designed to be representative of the United States (U.S.) supply merchandised in retail markets. Retail packages of raw beef heart, liver, kidney, tongue, honeycomb tripe, oxtail, and marrow bones were obtained from three different processing facilities (Texas, Nebraska, and Kansas) in the United States, to provide national representation of retail-ready beef offal items. Beef testicles were obtained from a single supplier in Colorado. Edible beef blood was obtained from a single processing facility in Pennsylvania.

Item name, description, and International Meat Purchasing Specifications (IMPS) identifier for each item are found in Table 1. From each of the three suppliers, beef heart, liver, kidney, tongue, honeycomb tripe, oxtail, and marrow bones were collected on two separate days, at least seven days apart. A minimum of four packages per collection date were procured of heart, liver, kidney, tongue, honeycomb tripe, and oxtail for a total of eight packages per item. Eight packages of vacuum sealed retail-ready marrow bone slices were obtained from the Kansas facility; femur bones were obtained from the Texas and Nebraska facilities and were sliced and vacuum sealed at the Colorado State University (CSU) Meat Lab to match the specifications of those collected from Kansas. Beef heart, liver, kidney, tongue, honeycomb tripe, oxtail, and marrow bones were maintained at 0–4 °C during transportation to the CSU Meat Laboratory. Upon arrival, packages were inspected for packaging integrity, and any packages with lack of a preserved seal were vacuum sealed immediately. All packages and containers were stored in a dark environment at 0–4 °C for 7 days post-production prior to being frozen at −20 °C for a minimum of 48 h, until dissection.

Three separate containers (4.0 kg each) of defibrinated edible beef blood were utilized, all from the Pennsylvania supplier. Three packages of testicles were used, all from a single supplier in Colorado. Beef blood and testicles were frozen to below 0 °C at each processing facility, maintained a temperature of below 0 °C during transport to the CSU Meat Laboratory, and stored at −20 °C upon arrival at the CSU Meat Laboratory until dissection.

#### 2.1.2. Dissection

Beef offal items were tempered in a single layer at 0–4 °C for 24–72 h, depending on item thickness, until the internal temperature reached 0–4 °C. After thawing, each individual sample was weighed with the packaging to the nearest 0.1 g, then removed from the package, and weighed to the nearest 0.1 g. The sample was blotted to remove any surface moisture and weighed again to the nearest 0.1 g. The internal temperature and start dissection time were recorded for each sample. The entire piece or pieces within a package were utilized for dissection. Post-dissection separable component weights and end dissection times and temperatures were recorded for each item. Dissections were performed using standard methods, including limited exposure to light, and use of powder-free nitrile gloves to protect nutrients from degradation. Dissections were performed by CSU personnel in a 5–7 °C environment using disposable stainless-steel scalpels (Integra Miltex, York, PA, USA) to yield separable components.

Separable components were defined as follows: separable lean tissue included any lean muscle or organ tissue, intramuscular fat, and light connective tissue deemed edible; external fat included adipose tissue located on the outer surface of the cut; internal fat, also known as seam fat, included adipose tissue deposited between lean tissue; refuse included all waste, comprised primarily of bone and heavy inedible connective tissue. For liquid items and items requiring no dissection (tripe, testicles, blood), separable lean tissue was used to describe the total sample. A yield tolerance of 97.0–100.0% was established prior to dissection. Any samples not meeting yield tolerance were removed from the study and replaced with a new sample of the same item, origin, and collection date. For dissected items, a total of three packages of each item from a single origin and collection date were used for homogenization, after meeting yield tolerance. Each of the separable components from each sample, excluding refuse, were homogenized individually immediately following dissection. Honeycomb tripe was procured following specification criteria of being practically devoid of external fat resulting in no dissection. Any tripe samples not meeting this criterion were trimmed at the CSU Meat Lab prior to homogenization. The testicles were devoid of fat, and the outer membrane of the testicle was removed at the processing facility; therefore, no dissection was necessary. Due to the liquid and homogeneous nature of the blood, the item was not altered prior to being frozen.

#### 2.1.3. Homogenization

For non-liquid items (heart, liver, kidney, tongue, oxtail, bone marrow, tripe, testicles), each separable component derived from a single package was homogenized; resulting in one lean sample per package, in addition to one external fat and one internal fat sample for each package, if present. Standard methods of homogenization were adhered to, including homogenizing with the use of powder-free nitrile gloves and in the absence of direct light to protect samples from contamination and nutrient degradation [11]. Separable lean tissue from each package was cut into 2.5 cm^3^ pieces and placed into a stainless steel strainer inside a stainless steel bowl containing liquid nitrogen until all pieces were completely frozen. The pieces were transferred into a 6.62-L Robot Coupe BLIXER 6V (Robot Coupe USA Inc., Ridgeland, MS, USA). Samples were blended for approximately 10 s at 1500 rpm and 30 s at 3500 rpm until a fine-powder consistency was reached. Immediately following homogenization, each sample was placed into a 710 mL Whirl-Pak bag using a stainless-steel spoon that was dipped in liquid nitrogen for 10 s before use. Each sample bag was placed into a −20 °C freezer immediately. External and internal fat samples were frozen following the same procedures as with lean tissue. After samples were frozen, samples were placed into a 3.79-L Robot Coupe BLIXER 4V (Robot Coupe USA Inc., Ridgeland, MS, USA) and blended into a finely powdered consistency under the same time and speed protocols as with lean homogenization. Fat samples were immediately placed into 532-mL Whirl-Pak bags using a stainless-steel spoon dipped in liquid nitrogen for 10 s before use. Sample bags were immediately placed into a −20 °C freezer.

Edible beef blood was homogenized using the same technique. One full container of blood, as procured, was blended in a 3.79-L stainless steel blender (Waring, Stamford, CT, USA). After blending, a stainless-steel ladle was drawn through the liquid from the bottom of the blender upward, and a 60 mL syringe (Medtronic, Minneapolis, MN, USA) was used to extract the liquid from the ladle. The syringe was used to create droplets that were dropped into a fine mesh strainer inside a stainless-steel bowl filled with liquid nitrogen. This procedure was repeated until at least 300 g of the sample was frozen as droplets. Samples were immediately placed into 710-mL Whirl-Pak bags using a stainless-steel spoon that was dipped in liquid nitrogen for 10 s before use. Each sample bag was immediately placed into a −20 °C freezer.

After samples were prepared each day, samples were double bagged and transferred from a −20 °C freezer into a −80 °C freezer until compositing and analysis occurred.

#### 2.1.4. Lean Compositing

For heart, liver, kidney, tongue, tripe, oxtail, and bone marrow, all homogenates of separable lean tissue of the same item and origin were combined in equal parts by weight to create three lean composites per item (one from each supplier). For testicles, three composites were created, one from each of three packages of product. Three composites of blood were created, one from each of three bottles of blood obtained. All compositing procedures occurred by combining lean homogenates, blending composites in a 6.62-L Robot Coupe BLIXER 6V (Robot Coupe USA Inc., Ridgeland, MS, USA), and aliquoting into Whirl-Pak bags in the presence of liquid nitrogen. All samples analyzed at an on-site laboratory were immediately placed back into a −80 °C freezer until analysis occurred. All samples analyzed at off-site laboratories were placed into a −80 °C freezer before being shipped in insulated boxes with dry-ice and gel ice packs via overnight shipping.

#### 2.1.5. Fat Compositing

For items containing separable fat (heart, kidney, tongue, oxtail), fat homogenates of the same item and fat type were combined in equal parts in weight. For oxtail, equal parts by weight of each fat type were then combined for a single composite. Oxtail was the only item containing external and internal fat. All compositing procedures occurred by combining fat homogenates, blending composites in a 3.79-L Robot Coupe BLIXER 4V (Robot Coupe USA Inc., Ridgeland, MS, USA), and aliquoting into Whirl-Pak bags in the presence of liquid nitrogen. All samples analyzed at an on-site laboratory were immediately placed back into a −80 °C freezer until analysis occurred. All samples analyzed at off-site laboratories were placed in a −80 °C freezer prior to being shipped in insulated containers with dry-ice and gel ice packs via overnight shipping.

### 2.2. Nutrient Analysis

Nutrient analysis occurred at United States Department of Agriculture (USDA) Agricultural Research Service (ARS)-approved laboratories, including CSU and commercial laboratories.

For beef heart, liver, kidney, and tongue, the following analyses were conducted: proximate analysis (protein, ash, moisture, fat), fatty acid profile, cholesterol, B vitamins (thiamin, niacin, riboflavin, pantothenic acid, vitamin B_6_, vitamin B_12_), vitamin A, vitamin E, vitamin D, 25-hydroxy vitamin D, vitamin K, calcium, copper, iron, magnesium, manganese, phosphorus, potassium, sodium, and zinc. The same nutrient analyses, excluding measurement of B vitamins, were performed for beef tripe, oxtail, bone marrow, testicles, and blood.

National Institute of Standards and Technology (NIST) standard reference material 1849a Adult/Infant Nutritional Supplement (Gaithersburg, MD, USA) and standard materials (Beech Nut Brand Beef and Chicken baby food, ground beef standard, pork and egg standard, beef bologna standard, and salmon standard) obtained from the Food Analysis Laboratory Control Center (Virginia Polytechnic Institute and State University; Blacksburg, VA, USA), were utilized to validate nutrient determinations to ensure the accuracy and precision of generated data among all laboratories. All standard materials were obtained from the Food Analysis Laboratory Control Center (FALCC; Virginia Polytechnic Institute and State University, Blacksburg, VA, USA). Ground beef and beef bologna standard materials were analyzed with each analysis group to ensure values existed within the acceptable range established by the FALCC for proximate analysis (protein, ash, fat, and dry matter). Mineral analyses were validated with use of NIST Adult/Infant Nutritional Supplement and beef bologna standard material. Beechnut beef baby food was used to validate thiamin, niacin, riboflavin, pantothenic acid, vitamin B_6_, and vitamin B_12_ assays. Beechnut chicken baby food was used for validation of the vitamin E assay. For cholesterol, vitamin B_12_, and fatty acid profile assays, ground beef standard material was utilized. Pork and egg standards were used to validate vitamin D, 25-hydroxy vitamin D analyses total thiamin, and vitamin K analyses. A salmon standard was utilized for validation of the amino acid profile and vitamin A assays. Chemical analyses were considered valid when the standard value generated was within the standard error of the certified value.

#### 2.2.1. Proximate Analysis

Proximate analysis was conducted to determine protein, ash, moisture, and fat content for all lean tissue composites for each item from each origin. Proximate analysis was conducted for fat composites for each item that contained separable fat (heart, kidney, tongue, oxtail).

##### Protein Analysis

Crude protein was determined following the AOAC Official Method 992.15 [13] using a nitrogen determinator (Leco TruSpec CN or Leco FP-2000; Leco Corporation, St. Joseph, MI and Rapid N Cube, Elementar, Hanau, Germany). The total percentage of nitrogen was multiplied by a factor of 6.25 to calculate percent protein. The protein content was determined at CSU.

##### Ash Analysis

The ash content was determined using the method described by AOAC 923.03 and 920.153 [14]. Approximately 1 g of sample was placed into a pre-weighed, dry crucible prior to placing the crucible into a box furnace (Thermolyne, Thermo Scientific, Waltham, MA, USA) at 600 °C for 18 h. The percent ash was calculated by dividing the ash weight by the initial sample weight and multiplying by 100. Ash analysis was conducted at CSU.

##### Moisture Analysis

The moisture content was determined using the oven drying method described in AOAC 950.46 and 934.01 [15]. Approximately 1 g of sample was weighted into aluminum tins prior to placing the tins into a forced air drying oven for 24 h at 100 °C. The percent moisture content was determined from the formula below. The moisture content was analyzed at CSU.

% Moisture = [(initial weight − dry weight)/initial weight] × 100

##### Fat Analysis

The fat content was determined using the chloroform:methanol method described by Folch, Lees, and Stanley (1957) [16]. Approximately 1 g of sample was homogenized in 2:1 chloroform:methanol solution prior to placement in an orbital shaker at room temperature for 20 min. The sample was filtered through ashless filter paper, and 4 mL of 0.9% NaCl was added before being refrigerated for 24 h. Upon phase separation of the filtrate, aspirated low phase content was placed into a pre-weighed scintillation vial and dried under N_2_ gas, followed by vial air drying under a hood for 2 h. Vials were placed into a forced air drying oven for 12 h at 100 °C. The total fat content was analyzed at CSU. The percent total fat was calculated from the formula:

% Total Fat = [((Total volume of chloroform:methanol)/10) × (final lipid weight/initial weight)] × 100

#### 2.2.2. Fatty Acid Analysis

Fatty acid methyl esters (FAMES) were prepared as described by Parks and Goins (1994) [17]. Analysis of FAMES occurred by liquid chromatography using an Agilent Model 6890 Series II (Avondale, PA, USA) gas chromatograph-fixed with a Series 7683 injector and flame ionization detector in addition to being equipped with a 100-m × 0.25-mm fused silica capillary column (SP-2560 Supelco Inc., Bellefonte, PA, USA). Fatty acid percentages were calculated based on the total FAME analyzed. Fatty acid analysis was conducted at CSU [18].

#### 2.2.3. Mineral Analysis

Mineral analyses were determined for Ca, Mg, K, Na, Fe, Zn, Cu, Mn, and P using inductively coupled plasma mass spectrometry methods described by the AOAC Official Methods 2011.19 and 993.14 [14] and USDA wet ash procedure. Mineral determination was conducted at Covance Laboratories (Madison, WI, USA).

#### 2.2.4. Cholesterol Analysis

Cholesterol analysis was performed using saponification, extraction, evaporation, and derivatization as described by AOAC Official Method 994.10 [14]. The cholesterol content was analyzed at Covance Laboratories (Madison, WI, USA).

#### 2.2.5. B-Vitamin Analysis

Analysis was conducted for thiamin, niacin, riboflavin, pantothenic acid, vitamin B_6_ and vitamin B_12_ using methods described as follows: total thiamin—AOAC Official Method 942.23, 953.17, 957.17; niacin—AOAC 944.13 and 960.46; riboflavin—AOAC 960.46 and 940.33; pantothenic acid—AOAC 945.74, 992.07, 960.46; vitamin B_6_—AOAC 961.15; vitamin B_12_—AOAC 952.20 and 960.46 [14]. Analysis of B-vitamins was conducted at Covance Laboratories (Madison, WI, USA).

#### 2.2.6. Fat-Soluble Vitamin Analysis

Vitamin A analysis was performed using HPLC with UV detection of retinol with external calibration, and internal standard recovery post analysis. This method is adapted from AOAC Official Method 2001.13 [14]. Vitamin A analysis was conducted by Craft Technologies Laboratory (Wilson, NC, USA). Vitamin D analysis was conducted by Covance Laboratories (Madison, WI, USA). Analysis was conducted for vitamin D_2_, D_3_, and 25-Hydroxy vitamin D_3_; the content was determined using the chromatography-mass spectrophotometry method described in AOAC Official Method 2011.11 [14]. Vitamin E analysis was conducted using high performance liquid chromatography (HPLC) with ultraviolet (UV) detection, with external calibration and internal standard recovery post-analysis. Vitamin E analysis was conducted by Craft Technologies Laboratory (Wilson, NC, USA). The vitamin K content was analyzed using HPLC with fluorescence detection after post-column reduction. Vitamin K analyses were performed at the Vitamin K Laboratory within the Jean Mayer USDA Human Nutrition Research Center on Aging (HNRCA) at Tufts University (Medford, MA, USA).

### 2.3. Data Analysis

Using R statistical software version 3.4.2 (R Core Team, 2017), the mean and standard error of the mean (SEM) of nutrient values were calculated from the three composites of each item for heart, liver, kidney, tongue, tripe, oxtail, and bone marrow. A single mean and SEM, representing a national average, were reported for the items listed above. Values from the three separate samples of testicles and blood were used to calculate a single mean and SEM value for each nutrient per item.

## 3. Results and Discussion

The beef offal items in this experiment are listed and described in Table 1.

### 3.1. Separable Components

Heart, liver, kidney, tongue, oxtail, and bone marrow were dissected to obtain individual separable components. The total grams of separable components for each item as well as the percentages of total pre-dissected weight comprised by each component are listed in Table 2. Internal fat was not present for heart, liver, or bone marrow; external fat was not present for liver, kidney, tongue, or bone marrow. Retail-ready packages of liver contained skinned and sliced portions, resulting in a high percentage of separable lean tissue with a small amount of refuse (thick blood vessels or connective tissue). Internal fat was present on the kidneys surrounding the blood vessels and ureter, which were trimmed flush with the kidney surface at the production facility. The intramuscular fat of the tongue muscle tissue was included in the separable lean tissue component, while the intermuscular fat was measured as internal fat, and refuse included the skin of the tongue. The percentages of separable lean tissue are relatively consistent with findings of Purchas, Wilkinson, and Carruthers (2015) [19], but some variation in fat and refuse values exists between the two studies. Fat content differences in particular are potentially attributable to the country of origin: New Zealand versus United States. Oxtail contained a significant amount of refuse due to the presence of bones (coccygeal vertebrae), resulting in a relatively low percentage of separable lean tissue. To obtain bone marrow, packages of sliced femur bones were obtained. The bone marrow was measured as separable lean tissue, while the bones made up the refuse for this item, which was a high percentage of the total weight.

### 3.2. Proximate Values

Mean and standard error of the mean for proximate composition, as well as cholesterol content, of separable lean components are presented in Table 3. Proximate and cholesterol analysis was also performed for fat samples and is displayed in Table 4.

#### 3.2.1. Protein

The protein content, on a percentage basis, ranged from approximately 10% to 20% for all items except bone marrow. These findings are consistent with protein content analysis of beef liver, heart, kidney, and tongue produced in other countries from varying production systems [5,20] Oxtail and liver had the greatest amount of protein on a percentage basis, both at above 19% of the total weight. Bone marrow contained a lower percentage of protein than the other items in the study, between 1% and 2%. Of note, some offal items included collagen, which is a structural protein with different characteristics than myofibrillar proteins that comprise the majority of skeletal muscle as is more often consumed in the U.S. The collagen content of beef tripe, as a proportion of total protein, has been determined to be about 20%, resulting in a unique amino acid pattern compared to skeletal muscle foods [21]. Although the individual amino acid content was not measured in this study, the protein digestibility of select offal items likely differs from traditionally consumed meat products since amino acid composition is a factor in determining the digestible indispensable amino acid score (DIAAS) [22].

#### 3.2.2. Fat

The fat content was analyzed for the separable lean component of each item; values were highly variable, with bone marrow having the highest percentage of total fat by a substantial margin at 77% of total weight. This falls within the range of total fat in bone marrow from caribou (72% to 84%), also a ruminant animal, which is consumed in some Northern regions of the world [6,23]. Tongue, tripe, and oxtail contained 11, 7, and 6% total fat, respectively; the mean values for all remaining items were below 5% total fat. An experiment aimed at determining the nutritional value of South African beef offal showed similar trends for the three items included in both studies [24]. The kidney contained the least fat with approximately 3% fat across the two experiments; a greater amount of fat and greater variation between studies (11% versus 18%) occurred for tongue. The heart fell between the kidney and tongue in both studies, with 3% and 7% of total weight comprised of fat in the current and South African study, respectively [24]. The percentage of fat within separable fat composites ranged from 43% to 49%, as shown in Table 4.

#### 3.2.3. Moisture

An inverse relationship has been shown in previous literature between the moisture content and fat content in meat products, specifically skeletal muscle [11,25,26]. Consistent with these findings, the items in the current study with the highest percentage of fat, bone marrow, and tongue had the least moisture. The moisture content of the fat composite samples ranged from 37% to 44%, with fat and moisture combined comprising over 80% of the total weight.

#### 3.2.4. Cholesterol

The greatest amount of cholesterol was detected in the kidney at 4 mg per g of lean tissue. The testicles and liver contained 2.19 mg and 2.57 mg per g, respectively. The heart, tripe, and blood were lower, between 1.16 mg and 1.28 mg per g. Containing the lowest amount of cholesterol were tongue and oxtail, both under 1 mg per g of separable lean; these values are comparable to the cholesterol levels found in beef skeletal muscle cuts, which have been reported in the 0.8–1 mg per g range [27]. A 2020 analysis of beef offal originating from South America indicated that tongue contained a higher amount of cholesterol (7.29 mg per g), while the cholesterol content of the kidney, liver, and heart were more consistent with current findings [28]. The discrepancy between cholesterol values for tongue may be a result of dissection in the current study to produce a separable lean component for analysis, rather than analyzing the complete tongue as a single item. It is of interest to note that the most recent editions of the Dietary Guidelines for Americans did not provide a quantitative upper limit for dietary cholesterol as evidence suggests a lack of correlation between dietary and blood cholesterol levels despite previous debate amongst the nutrition community [2,3,29].

### 3.3. Vitamin Analysis

The results of vitamin A, D, E, K, and 25-Hydroxy vitamin D analysis for separable lean tissue of the offal items are presented in Table 5 and Table 6. While four forms of tocopherols (alpha, beta, delta, gamma) were analyzed, only alpha-tocopherol analysis results are presented as the other vitamin E compounds were below the limit of detection (0.05 mcg/g) with the exception of gamma-tocopherol in blood (Mean: 0.11 mcg/g, SEM: 0.02 mcg/g). The vitamin D_2_ levels were below the limit of detection (0.001 mcg/g) for all separable lean samples and all fat composites. Vitamin D_3_ was below the limit of detection (0.001 mcg/g) for at least one sample of each separable lean composite except kidney (Mean: 0.003 mcg/g, SE: 0.0007), tongue (Mean: 0.001 mcg/g, SE: 0.0001), and bone marrow (Mean: 0.009 mcg/g, SE: 0.0006). The B-vitamin content results, for the four items analyzed, are found in Table 7. For fat composites, vitamin analysis results are shown in Table 4. To provide greater clarity, discussion in this section focuses on those vitamins that contribute significantly to recommended daily intake amounts.

#### 3.3.1. Vitamin A

The vitamin A content was analyzed for all items. The liver contained the greatest amount of vitamin A, with a mean of 37 mcg/g of separable lean, or 3702 mcg per 100 g (12,340 International Units), which is 247% of the Daily Value (DV) for vitamin A. The separable lean component from other items, as well as the fat composites, contained considerably less vitamin A: under 1 mcg/g. These results support previous claims that beef liver is an excellent source of vitamin A. However, the amount of vitamin A in liver previously reported in FoodData Central was higher, at 26,088 International Units (IU) [6]. The vitamin A content in the liver can be influenced by production method and animal diet, among other factors, which may explain the variation in values [30,31]. The Tolerable Upper Intake level for vitamin A, as established by the Food and Nutrition Board of the Institute of Medicine, is 3000 mcg per day; this value represents the highest amount of a nutrient that most people may consume to result in no adverse health risks [32]. However, much higher consistent consumption levels of vitamin A are considered generally toxic: greater than 25,000 IU per day for six years or greater than 100,000 IU per day for six months [21]. Nonetheless, it is important to have current vitamin A content information for liver and other offal items to allow for appropriate nutrition recommendations for a range of individuals and populations.

#### 3.3.2. Vitamin K

Both phylloquinone (vitamin K_1_) and ten forms of menaquinones (vitamin K_2_) were analyzed for all samples in this study. Bone marrow contained the highest level of vitamin K_1_: 166 ng/g of separable lean, representing 14% of the DV. The vitamin K_1_ content in other items ranged from 0.5 ng/g to 9.7 ng/g. The fat composite samples contained between 27.3 ng/g to 69.1 ng/g of phylloquinone. Menaquinones were present in all samples analyzed, with menaquinone-4 and menaquinones-9, -10, -11, -12, and -13 present in the largest amount in separable lean. Menaquinone-5 was not detectable in any of the items. Menaquinones-4, -9, -10, -11, -12, and -13 were present in the fat samples, while the other forms were not detectable.

Current dietary reference values (DRV) for vitamin K are solely based on the phylloquinone form, which is derived mainly from plant sources and plays a role in blood coagulation. However, menaquinones, which are found in higher amounts in animal products and fermented foods, have been studied recently in regard to their effect on human health. A study conducted by Beulens and colleagues found an association between vitamin K_2_ intake and decreased coronary calcification, suggesting that it may be able to play a role in preventing cardiovascular disease [33]. Studies have also shown vitamin K_2_ to be associated with improved bone quality and decreased incidence of bone fractures [34,35,36,37]. The research on dietary needs, bioavailability, and health impacts of vitamin K_2_ are limited, but this area warrants further investigation.

#### 3.3.3. B-Vitamins

The heart, liver, kidney, and tongue were evaluated for their B-vitamin content. Riboflavin was present in all 4 items at levels above 20% of the DV for this nutrient, ranging from 3.6 mcg to 33.8 mcg per g of separable lean. Both the niacin and pantothenic acid content were highest in the liver at 141 mcg/g and 57 mcg/g, respectively, providing over half of the DV for both nutrients in 100 g of separable lean tissue. Other items contained between 39 and 63 mcg/g of niacin and between 5 and 22 mcg/g of pantothenic acid on a separable lean basis. The pyridoxine hydrochloride form of vitamin B_6_ was present at levels ranging from 2 to 10 mcg per g of separable lean in the 4 items analyzed. Vitamin B_12_ was also present in each of the items, with content between 0.05 and 0.85 mcg/g, providing at least 90% of the DV per 100 g serving. The liver contained the greatest amount of vitamin B_12_ (0.85 mcg/g), with the observed mean higher than previously published values [6,10]. Thiamin was present in the lowest quantities with less than 1 mcg/g of separable lean tissue.

Although numerical values for several of the B-vitamins appeared to be minimal, daily requirements of these vitamins are low compared to many other nutrients. Consequently, the B-vitamin content of these offal items contributes a significant amount to the DV. Skeletal muscle and other animal products are considered to be a valuable source of B-vitamins, especially vitamin B_12_ [38], and these results provide further evidence that offal can be included in this claim. In fact, researchers recently modeled the contribution of meat to global nutrient availability and found that edible offal across species provides 31% of the Vitamin B_12_ available for consumption, as well as 5% of riboflavin and 3% of pantothenic acid [10]. Tolerable upper intake levels are not determined for riboflavin, pantothenic acid, or vitamin B_12_. However, the upper intake value for niacin is 35 mg/day and for vitamin B_6_ is 100 mg/day [39], which are significantly greater than the amounts contained in a 100 g portion any of the items in the study.

### 3.4. Inductively Coupled Plasma Mineral Analysis

The results of mineral analyses for the separable lean tissue of each item are presented in Table 8. The calcium content was highest in the bone marrow samples, contributing over 64% of the DV per 100 g of bone marrow. Bone marrow from other ruminant animals has been shown to also have elevated levels of calcium, especially compared to skeletal muscle and organ meat [23]. The liver samples contained the largest amount of copper by a large margin with 119 mcg/g of separable lean tissue, followed by heart and kidney with 3.7 mcg/g and 4.8 mcg/g, respectively. These results align with previous research findings, despite differing production systems [20]. The amount of copper in 100 g of liver (11,900 mcg) relates to nearly 600% of the DV for copper, exceeding the tolerable upper limit of 10,000 mcg [32]. While gastrointestinal discomfort may occur at a copper intake as low as 5000 mcg per day, the level at which liver damage may occur is more difficult to establish [40]. Research suggests that a daily copper intake of 10,000 mcg for multiple weeks would not result in toxicity in individuals with normal ability to maintain copper homeostasis [41]. Nonetheless, it may be important to consider copper levels in liver especially for those consuming this product as part of their regular diet.

Iron was present at notable levels in the heart, liver, kidney, tongue, oxtail, and blood, as is also indicated in prior research despite some discrepancy among values, particularly for the kidney [5]. The blood contained the most iron: 491.33 mcg/g, providing more than 200% of the DV in a 100-g portion. It would be important to consider the form in which blood is consumed, as it is typically used as an ingredient and therefore eaten in smaller quantities. However, the high iron content of blood could potentially be taken advantage of for nutritional purposes, especially as blood has previously been tested as a fortification medium for other nutrients [42]. Levels of manganese were relatively low in all samples, with the liver and kidney containing the most. Manganese values were consistent with previously published data for liver and kidney from beef raised in an organic production system [20]. However, the Mn content in beef heart from the same study was reported at higher levels than observed in the current analysis, more consistent with the liver content [20]. Phosphorus was present in all samples and provided from 15% to 36% of the DV in 100 g portions of liver, kidney, tongue, oxtail, bone marrow, and testicles, with the other items containing lesser amounts. The phosphorus content of the heart, liver, kidney, and tongue aligns with data reported in prior studies [5]. Zinc levels were lowest in blood and testicles but were sufficient to contribute 10–35% of the DV per 100 g of each of the other items.

### 3.5. Fatty Acid Profile

The fatty acid profiles of fat samples for the heart, kidney, tongue, and oxtail are presented in Table 9. The fatty acid profiles of separable lean tissue are presented in Table 10. The most prevalent fatty acid (as a percentage of total fatty acids in each sample) for both lean tissue and fat samples was oleic acid (C18:1c9), a monounsaturated fatty acid (MUFA) that is commonly associated with olive oil. Monounsaturated fatty acids have been associated with decreased LDL and total cholesterol and increased HDL cholesterol when they replace other macronutrients in the diet, such as saturated fatty acids and carbohydrates [43]. The percentage of MUFA in the separable lean component of offal items ranged from 17 to 45% of the total fatty acid composition. The high end of this range is similar to the percentage of MUFA previously reported for raw beef muscle cuts from the loin and round of 40–47% [11]. In the fat samples from the heart, kidney, tongue, and oxtail, MUFA made up 40–42% of the total fatty acid matrix. Data available for the monounsaturated fatty acid content of the heart, liver, kidney and tongue, as a percent of total fat, is consistent with values observed in the current study with variations ranging from 1.5% for liver to 3.4% for tongue [5].

Following oleic acid, stearic acid (C18:0) and palmitic acid (C16:0) made up the next highest percentage of total fatty acids for both lean tissue and fat samples. Research has shown that stearic acid, unlike some saturated fatty acids, does not have a negative impact on serum cholesterol levels [43,44,45]. In total, saturated fatty acids composed about 35–53% of the total fatty acid content in the separable lean component of all items in the study. As with monounsaturated fat, the percent of saturated fat for the heart, liver, kidney, and tongue is comparable with previous research findings of Latoch et al.; the largest discrepancy was for kidney, which differed by 4.68% [5].

The polyunsaturated fatty acid linoleic acid (C18:2) was the fourth most prevalent fatty acid in the samples. Linoleic acid made up a larger percentage of the fatty acid profile in the separable lean tissue of the heart, liver, kidney, and blood compared to other items. Linoleic acid must be obtained from dietary sources, as the body cannot synthesize this fatty acid. Conjugated linoleic acid (CLA) was also present in the offal items. Conjugated linoleic acid is a term used that refers to the isomers of linoleic acid; this group of polyunsaturated *trans* fatty acids are found naturally in food products derived from ruminant animals. Previous studies have indicated that CLA may have anticarcinogenic properties [46,47], and researchers continue to investigate the impact of CLA on various aspects of human health [48]. However, additional evidence is necessary to determine whether positive outcomes can be attributed to CLA intake.

### 3.6. Extra-Labeling Claims

The U.S. Food and Drug Administration (FDA) regulates the use of nutrient label claims on food products and provides guidelines for the requirements of these claims (9 C.F.R. 317.354). The claim “Good Source”, which is equivalent to “Contains” and “Provides”, can be used on a label if the product contains 10–19% of the DV or Recommended Daily Intake (RDI) per RACC (reference amount customarily consumed) for that nutrient. To include a claim of “Excellent Source”, “High”, or “Rich In”, the food must contain at least 20% of the DV or RDI per RACC for that nutrient. Each of the items evaluated in this study qualified for at least one “Good Source” or “Excellent Source” nutrient labeling claim. The following results were based on separable lean tissue only and are depicted in Table 11.

The heart was an excellent source of protein, riboflavin, niacin, vitamin B_12_, and iron; it was a good source of pantothenic acid, vitamin B_6_, copper, and zinc. The liver was an excellent source of protein, vitamin A (retinol), riboflavin, pantothenic acid, vitamin B_6_, niacin, vitamin B_12_, copper, iron, phosphorus, and zinc; it was a good source of manganese. The kidney was an excellent source of protein, riboflavin, pantothenic acid, vitamin B_6_, vitamin B_12_, copper, iron, and phosphorus; it was a good source of zinc. The tongue was an excellent source of protein, riboflavin, niacin, vitamin B_12_, and zinc; it was a good source of vitamin B_6_, iron, and phosphorus. The oxtail was an excellent source of protein and zinc; it was a good source of iron and phosphorus. The tripe was an excellent source of protein and a good source of zinc. The bone marrow was an excellent source of calcium and phosphorus; it was a good source of vitamin K_1_. The testicles were an excellent source of protein and phosphorus. The blood was an excellent source of protein and iron. The beef offal items in this study provide significant levels of protein, along with food items comprised of beef skeletal muscle. Additionally, these products are rich in other micronutrients as described above. Offal items are often more affordable than other beef cuts, particularly in the U.S., suggesting that these items can be valuable in providing essential nutrients to resource-limited populations or individuals.

### 3.7. Summary and Significance

Nutritional analyses revealed the availability of essential nutrients in beef offal. Although some discrepancies exist, the values observed as part of the current study are reasonably consistent when compared to data published previously. Of the items included, the liver is a particularly nutrient-dense product, as well as the heart, kidney, and tongue. As could be expected due to the products originating from cattle, protein and B-vitamin content were among the most substantial as related to human dietary needs and recommendations.

Offal items may become more highly demanded as the growing global population experiences an increased need for nutrient-rich food sources. The broader category of meat, across species and products, is documented to disproportionally contribute to nutrient availability on a global scale, as compared to other food products [10]. Offal contains many of the same vitamins, minerals, and macronutrients as skeletal muscle but may come at an economic advantage; opportunity exists for these products to fill nutrient gaps through direct consumption or other methods such as fortification or supplementation.

The nutrient content is one factor people consider in making food-related choices, yet there are undoubtedly many others. Consumer preferences for offal items have been evaluated, and research highlights a general aversion to these products [8,9,49]. However, among populations who regularly consume offal items, related choices are highly influenced by an item’s nutritional value as well as status as a delicacy [50]. Further research suggests pathways for increasing the acceptability of offal products, including communicating benefits, showcasing usage in alignment with typical culinary practices, and increasing familiarity of the products [8,49].

## 4. Conclusions

This study provided analytically derived nutrient information for U.S. beef offal items including heart, liver, kidney, tongue, honeycomb tripe, oxtail, bone marrow, testicles, and blood. These data are critical for use by researchers and nutrition professionals, who influence the general population. According to percentages calculated from the separable lean component only, each of the items included in the study qualified for a “Good Source” or “Excellent Source” extra labeling nutrient claim for at least one nutrient, as defined by the FDA. The results suggest that offal items can be beneficial in providing essential vitamins and minerals as a component of a healthy diet. Consumption of these items is more prevalent in developing countries but also occurs frequently in regions of the United States. Having comprehensive nutrient data will aid in understanding the contribution of beef offal to the diet of certain populations. Findings from this study suggest that edible offal products provide potential for increasing the nutrient density of diets that are deficient in protein and certain micronutrients. Additionally, the results may present an opportunity for food technologists to utilize offal items in developing new food or supplement products that could contribute to the nutritional needs of individuals and populations.

## Figures and Tables

**Table 1:** Description of U.S. beef offal items and International Meat Purchase Specifications (IMPS) numbers.

Item Name	Description	IMPS Number ^1^
Beef heart, cap off	Cap removed (including auricles, arteries, gristly material); bone removed	720
Beef liver, sliced	Fabricated from skinned, deveined, defatted liver; sliced to 0.25–0.5 inches thick	702
Beef kidney	Blood vessels, pizzle cord, and ureter trimmed flush with kidney surface; capsule membrane surrounding kidney removed	722
Beef tongue, short cut	Tongue removed directly behind base of hyoid bone; hyoid bones, glandular tissue and trachea removed; epiglottis and major blood vessels trimmed flush with surface	716
Beef honeycomb tripe, scalded	Reticulum; dark internal intestinal lining removed; bleached (scalded)	726
Beef oxtail, segmented	Skinned tail; removed at juncture of second and third coccygeal vertebrae; external fat trimmed to no more than 25 inches; cut into segments	721
Beef bone marrow	Marrow extracted from femur bones, knobs removed from ends; cut to approximately 1 inch slices	-
Beef testicles	Testicles; cremasteric muscle and spermatic cord trimmed flush with surface; membrane surrounding testicle removed	-
Beef blood	Defibrinated edible blood, salt added; bottled	-

^1^ IMPS not defined for bone marrow, testicles, or blood.

**Table 2:** Mean and standard error of the mean of separable components derived from raw U.S. beef offal items expressed as grams (g) and as a percentage of pre-dissected weight.

	Separable Tissue ^1^		External Fat ^2^		Internal Fat ^3^		Refuse ^4^	
	(g)	(%)	(g)	(%)	(g)	(%)	(g)	(%)
Item								
Heart	650.58 ± 135.20	88.23 ± 3.27	60.96 ± 24.81	7.95 ± 2.27	0	0	19.32 ± 10.20	3.12 ± 1.69
Liver	480.99 ± 39.23	95.97 ± 1.14	0	0	0	0	11.39 ± 3.77	2.37 ± 0.91
Kidney	678.34 ± 151.09	85.22 ± 2.57	0	0	92.12 ± 35.36	10.99 ± 2.74	13.23 ± 8.32	2.19 ± 1.48
Tongue	1014.77 ± 84.01	73.32 ± 2.28	0	0	139.40 ± 33.98	9.82 ± 1.76	220.62 ± 37.72	15.82 ± 1.96
Tripe ^5^	1102.93 ± 64.94	100.00	0	0	0	0	0	0
Oxtail	346.21 ± 34.81	34.82 ± 2.50	136.24 ± 20.40	13.82 ± 2.16	53.44 ± 19.39	5.30 ± 1.84	444.64 ± 47.76	44.76 ± 3.72
Bone Marrow	198.68 ± 20.89	16.30 ± 0.84	0	0	0	0	1015.34 ± 102.1	83.88 ± 7.69
Testicles ^5^	2203.87 ± 10.13	100.00	0	0	0	0	0	0

^1^ Separable lean tissue weight (g) includes all lean muscle and organ tissue. Separable lean, %: [separable lean tissue (g)/pre-dissection cut weight (g)] × 100. ^2^ External fat weight (g) includes all fat located on the outer surface of the item. External fat, %: [external fat (g)/pre-dissection cut weight (g)] × 100. External fat was not present for liver, kidney, tongue, or bone marrow. ^3^ Internal fat weight (g) includes any fat which lies between lean tissue. Internal fat, %: [internal fat (g)/pre-dissection cut weight (g)] × 100. Internal fat was not present for heart, liver, or bone marrow. ^4^ Refuse weight (g) includes all bone and heavy connective tissue (including tongue skin). Refuse, %: [refuse (g)/pre-dissection (g)] × 100. ^5^ Tripe and testicles were not dissected due to the homogeneous nature of the products; the entire item was considered separable lean tissue.

**Table 3:** Mean and standard error of the mean of proximate values (% protein, % total fat,% ash, and % moisture) and cholesterol content of 100 g of separable lean tissue ^1^ from raw U.S. beef offal items.

Cut	Protein (%)	Total Fat (%)	Ash (%)	Moisture (%)	Cholesterol mg/g
Heart	17.48 ± 0.19	3.21 ± 0.18	1.19 ± 0.07	78.70 ± 0.36	1.16 ± 0.01
Liver	19.57 ± 0.16	4.50 ± 0.12	1.46 ± 0.04	71.47 ± 0.08	2.57 ± 0.04
Kidney	17.41 ± 0.53	3.31 ± 0.26	1.14 ± 0.08	78.79 ± 0.31	4.00 ± 0.10
Tongue	17.42 ± 0.5	11.20 ± 0.15	1.00 ± 0.08	69.61 ± 0.08	0.87 ± <0.01
Tripe	12.15 ± 0.4	7.23 ± 0.48	0.52 ± 0.02	78.78 ± 0.44	1.24 ± 0.06
Oxtail	19.91 ± 0.22	6.45 ± 0.07	0.82 ± 0.03	71.68 ± 0.34	0.67 ± 0.02
Bone Marrow	1.25 ± 0.08	77.09 ± 0.23	0.62 ± 0.06	11.51 ± 0.31	1.28 ± 0.01
Testicles	10.82 ± 0.04	2.91 ± 0.11	1.20 ± 0.03	86.18 ± 0.05	2.19 ± 0.02
Blood	18.85 ± 0.31	1.15 ± 0.02	3.10 ± 0.08	77.38 ± 0.06	1.57 ± 0.01

^1^ Separable lean tissue includes all lean muscle and organ tissue.

**Table 4:** Proximate values and nutrient content of fat from raw U.S. beef offal items as a single national composite per item ^1^.

	Heart Fat	Kidney Fat	Tongue Fat	Oxtail Fat ^2^
Component, units				
Protein, %	8.56	8.03	8.79	7.50
Fat, %	46.33	43.22	49.28	49.28
Ash, %	0.38	0.39	0.40	0.28
Moisture, %	38.83	37.22	43.70	38.55
Nutrient, units				
Cholesterol, mg/g	1.00	1.32	1.27	0.92
Retinol (Vitamin A), mcg/g	0.37	0.38	0.10	0.27
Vitamin D_3_, mcg/100 g	0.31	0.46	0.29	0.26
25-Hydroxy Vitamin D, mcg/100 g ^3^	0.57	1.21	0.73	0.53
Alpha-Tocopherol (Vitamin E), mcg/g	9.25	10.56	6.86	6.95
Phylloquinone (Vitamin K_1_), mcg/g	37.60	69.10	28.80	27.30
Vitamin K_2_^4^				
Menaquinone-4, ng/g	300.00	273.00	344.50	183.50
Menaquinone-9, ng/g	11,900.00	14,200.00	13,500.00	14,370.00
Menaquinone-10, ng/g	3826.00	2043.00	3568.00	4255.00
Menaquinone-11, ng/g	48,540.00	38,720.00	29,269.00	27,200.00
Menaquinone-12, ng/g	88.10	132.50	64.20	54.00
Menaquinone-13, ng/g	0.00	0.00	0.00	0.00
Calcium, mcg/g	<38.50 ^5^	41.30	43.10	58.50
Copper, mcg/g	1.36	1.06	0.66	<0.388
Iron, mcg/g	20.60	14.40	14.60	7.91
Magnesium, mcg/g	99.40	59.00	90.90	72.30
Manganese, mcg/g	<0.194	0.23	0.21	<0.194
Phosphorus, mcg/g	830.00	605.00	858.00	624.00
Potassium, mcg/g	1290.00	895.00	1440.00	1160.00
Sodium, mcg/g	486.00	747.00	510.00	565.00
Zinc, mcg/g	6.90	6.96	10.40	13.70

^1^ The single fat composite for each item (heart, kidney, tongue, oxtail) included equal g of fat from each of the three respective individual items from each of three suppliers. ^2^ Oxtail fat is an equal composite of external and internal fat (each composited as stated above); both types were present only for this item. ^3^ Values for Vitamin D_3_ and 5-Hydroxy Vitamin D are reported as mcg/100 g due to the small amount present in samples. ^4^ Menaquinone-5, -6, -7, -8 were not detectable for any fat samples. ^5^ Values preceded by “<” were below the limit of quantification.

**Table 5:** Mean and standard error of the mean vitamin values from separable lean tissue ^1^ from six ^2^ raw U.S. beef offal items.

Nutrient, Units	Heart	Liver	Kidney	Tongue	Tripe	Oxtail
Retinol (Vitamin A), mcg/g	0.02 ± 0.00	37.02 ± 9.39	0.25 ± 0.11	0.05 ± 0.02	0.03 ± 0.01	0.03 ± 0.02
25-Hydroxy Vitamin D, mcg/100 g ^3^	0.52 ± 0.11	0.62 ± 0.07	1.26 ± 0.08	0.51 ± 0.03	0.57 ± 0.07	0.29 ± 0.03
Alpha-Tocopherol (Vitamin E), mcg/g	8.18 ± 0.83	4.72 ± 0.36	4.36 ± 0.75	4.20 ± 0.15	1.80 ± 0.22	2.96 ± 0.50
Phylloquinone (Vitamin K_1_), ng/g	3.17 ± 0.23	9.67 ± 2.46	1.27 ± 0.09	8.83 ± 2.46	6.43 ± 0.78	3.43 ± 0.58
Vitamin K_2_^4^						
Menaquinone-4, ng/g	16.47 ± 2.28	-	45.37 ± 13.68	88.27 ± 13.65	127.83 ± 13.17	47.80 ± 2.29
Menaquinone-6, ng/g	4.70 ± 1.06	31.00 ± 3.57	-	-	-	-
Menaquinone-7, ng/g	-	77.87 ± 13.11	-	-	-	-
Menaquinone-8, ng/g	-	37.33 ± 5.21	-	-	-	-
Menaquinone-9, ng/g	34.57 ± 4.08	71.13 ± 21.90	11.77 ± 0.33	3518.33 ± 281.90	1609.50 ± 495.08	1612.00 ± 99.14
Menaquinone-10, ng/g	79.07 ± 29.57	67.37 ± 15.28	0.00	1306.17 ± 319.70	497.60 ± 194.81	431.63 ± 72.14
Menaquinone-11, ng/g	1897.33 ± 424.33	1340.67 ± 181.80	1104.83 ± 70.84	10,224.33 ± 1370.37	14,229.00 ± 2916.51	7044.33 ± 521.38
Menaquinone-12, ng/g	5.67 ± 0.70	537.83 ± 55.23	15.03 ± 1.40	16.10 ± 4.75	38.40 ± 7.47	19.77 ± 5.36
Menaquinone-13, ng/g	14.27 ± 1.41	1707.33 ± 126.14	42.33 ± 6.47	-	7.90 ± 3.95	-

^1^ Separable lean tissue includes all lean muscle and organ tissue. ^2^ Vitamin analysis results of other offal items in the study are presented in a separate table. ^3^ Values for 5-Hydroxy Vitamin D are reported as mcg/100 g due to the small amount present in samples. ^4^ A single dash indicates that the nutrient was below the limit of detection; the limit of detection was not defined.

**Table 6:** Mean and standard error of the mean vitamin values from separable lean tissue ^1^ from three ^2^ raw U.S. beef offal items.

Nutrient, Units	Bone Marrow	Testicles	Blood
Retinol (Vitamin A), mcg/g	0.48 ± 0.10	0.05 ± 0.02	0.07 ± 0.02
25-Hydroxy Vitamin D, mcg/100 g	0.45 ± 0.02	0.47 ± 0.04	2.30 ± 0.08
Alpha-Tocopherol (Vitamin E), mcg/g	8.50 ± 3.61	20.23 ± 3.23	2.21 ± 0.12
Phylloquinone (Vitamin K_1_), ng/g	166.00 ± 36.87	7.93 ± 0.43	0.53 ± 0.03
Vitamin K_2_^3^			
Menaquinone-4, ng/g	338.00 ± 43.42	335.83 ± 26.28	0.00
Menaquinone-6, ng/g	-	2.27 ± 2.27	0.73 ± 0.15
Menaquinone-7, ng/g	-	10.57 ± 5.43	1.50 ± 0.00
Menaquinone-8, ng/g	-	-	-
Menaquinone-9, ng/g	23,000.00 ± 1249.00	-	-
Menaquinone-10, ng/g	6124.67 ± 1453.62	-	9.53 ± 0.73
Menaquinone-11, ng/g	52,866.67 ± 2273.27	790.67 ± 60.32	27.40 ± 2.59
Menaquinone-12, ng/g	186.00 ± 76.53	9.37 ± 1.53	17.30 ± 0.35
Menaquinone-13, ng/g	-	94.53 ± 4.45	26.83 ± 0.93

^1^ Separable lean tissue includes all lean muscle and organ tissue. ^2^ Vitamin analysis results of other offal items in the study are presented in a separate table. ^3^ A single dash indicates that the nutrient was below the limit of detection; the limit of detection was not defined.

**Table 7:** Mean and standard error of the mean of B-vitamin values from separable lean tissue ^1^ from raw U.S. beef offal items.

B-Vitamin, Units	Heart	Liver	Kidney	Tongue
Thiamin (Vitamin B_1_), mcg/g	0.36 ± 0.01	0.18 ± 0.01	0.36 ± 0.01	0.09 ± <0.01
Thiamin Hydrochloride (Vitamin B_1_), mcg/g	0.46 ± 0.01	0.23 ± 0.01	0.45 ± 0.02	0.12 ± 0.00
Riboflavin (Vitamin B_2_), mcg/g	11.4 ± 0.04	33.8 ± 0.28	29.1 ± 0.40	3.60 ± 0.36
Niacin (Vitamin B_2_), mcg/g	45.13 ± 1.45	141.33 ± 4.37	62.67 ± 1.10	39.47 ± 3.99
Pantothenic Acid (Vitamin B_5_), mcg/g	12.63 ± 4.57	56.93 ± 4.66	22.13 ± 8.82	5.03 ± 2.24
D Calcium Pantothenate (Vitamin B_5_), mcg/g	13.77 ± 4.95	61.87 ± 5.05	24.03 ± 9.58	5.47 ± 2.46
Pyridoxine Free Base (Vitamin B_6_), mcg/g	2.92 ± 0.16	7.87 ± 0.12	5.88 ± 0.99	2.11 ± 0.08
Pyridoxine Hydrochloride (Vitamin B_6_), mcg/g	3.55 ± 0.20	9.56 ± 0.14	7.15 ± 1.21	2.57 ± 0.10
Vitamin B_12_, mcg/g	0.12 ± 0.03	0.85 ± 0.12	0.43 ± 0.03	0.05 ± <0.01

^1^ Separable lean tissue includes all lean muscle and organ tissue.

**Table 8:** Mean and standard error of the mean values of mineral content from separable lean tissue ^1^ from raw U.S. beef offal items.

Cut	Calcium, mcg/g	Copper, mcg/g	Iron, mcg/g	Magnesium, mcg/g	Manganese, mcg/g	Phosphorous, mcg/g	Potassium, mcg/g	Sodium, mcg/g	Zinc, mcg/g
Heart	43.23 ± 1.39	3.70 ± 0.05	46.40 ± 1.01	223.33 ± 2.03	0.34 ± 0.01	2000.00 ± 28.87	2610.00 ± 43.59	951.33 ± 36.61	18.03 ± 0.37
Liver	40.23 ± 0.55	119.47 ± 25.78	51.53 ± 1.45	185.67 ± 1.86	2.60 ± 0.10	3550.00 ± 30.55	2910.00 ± 26.46	616.00 ± 10.58	38.90 ± 2.86
Kidney	84.97 ± 2.64	4.80 ± 0.22	52.13 ± 2.66	165.33 ± 0.88	1.09 ± 0.03	2336.67 ± 6.67	2340.00 ± 69.28	1843.33 ± 52.07	20.73 ± 0.70
Tongue	46.67 ± 1.05	1.09 ± 0.07	22.97 ± 0.98	180.67 ± 5.21	- ^3^	1500.00 ± 40.41	2523.33 ± 72.65	784.33 ± 19.89	34.30 ± 0.67
Tripe	181.00 ± 29.31	<0.50 ^2^ ± 0.07	5.21 ± 0.11	106.23 ± 8.76	<0.542^2^ ± 0.23	623.67 ± 16.50	876.67 ± 43.38	929.33 ± 135.93	15.83 ± 0.49
Oxtail	86.27 ± 5.80	0.95 ± 0.19	19.07 ± 1.27	193.33 ± 5.04	-	1500.00 ± 40.41	2456.67 ± 59.25	1106.67 ± 33.83	53.73 ± 2.79
Bone Marrow	6450.00 ± 4175.82	<0.42 ^2^ ± 0.01	6.75 ± 0.41	<126.972^2^ ± 72.07	-	2982.00 ± 1862.48	<197.33^2^ ± 1.76	467.33 ± 123.11	2.92 ± 1.23
Testicles	74.53 ± 7.17	0.82 ± 0.11	16.07 ± 0.90	132.67 ± 5.61	0.32 ± 0.02	2096.67 ± 85.11	3013.33 ± 121.29	1166.67 ± 44.85	14.00 ± 0.55
Blood	64.33 ± 0.94	1.06 ± 0.14	491.33 ± 6.01	<39.702^2^ ± 0.17	-	191.67 ± 2.33	549.00 ± 4.00	11,233.33 ± 218.58	2.97 ± 0.06

^1^ Separable lean tissue includes all lean muscle and organ tissue. ^2^ At least one of the composite values used to calculate the mean was below the limit of detection for the corresponding nutrient. ^3^ A single dash indicates that manganese was below the limit of detection (limit of detection: 0.20 mcg/g).

**Table 9:** Fatty acid profile of raw U.S. beef offal item fat samples analyzed as a single composite per item ^1^, as a percentage of total fatty acids (g/100 g fat).

Fatty Acid Name/Group		Heart Fat	Kidney Fat	Tongue Fat	Oxtail Fat ^2^
Capric acid (C10:0)		0.02	0.00	0.00	0.00
Lauric acid (C12:0)		0.05	0.00	0.00	0.00
Myristic acid (14:0)		0.59	0.44	0.40	0.39
Palmitic acid (16:0)		24.63	21.94	22.05	23.14
Margaric acid (17:0)		1.32	1.30	1.28	1.23
Stearic acid (18:0)		20.71	23.91	23.04	24.09
Arachidic acid (20:0)		0.02	0.01	0.08	0.03
Lignoceric acid (24:0)		0.06	0.08	0.03	0.04
**Total SFA**		**47.40**	**47.68**	**46.88**	**48.92**
Lauroleic acid (12:1 n3)		0.01	0.00	0.00	0.00
C14:1		0.49	0.24	0.33	0.26
Palmitoleic acid (16:1 n7)		3.24	4.72	4.07	3.92
C17:1		1.12	1.05	1.04	1.05
Oleic acid (C18:1 c9)		35.91	34.62	35.34	34.38
C18:1c11		1.11	1.02	1.01	1.07
Eicosenoic acid (C20:1)		0.12	0.09	0.10	0.12
**Total MUFA**		**42.00**	**41.74**	**41.89**	**40.80**
Linolelaidic acid (C18:2)		5.06	4.54	5.14	4.16
Linolenic acid (C18:3)		0.24	0.21	0.20	0.26
Arachidonic acid (C20:4)		0.39	0.31	0.38	0.39
**Total PUFA**		**5.69**	**5.06**	**5.72**	**4.81**
C18:1t6-8		0.61	0.53	0.63	0.55
Elaidic acid (C18:1 t9)		0.49	0.42	0.45	0.45
C18:1t10		2.38	3.05	2.90	2.99
Vaccenic acid (C18:1 t11)		1.05	1.07	1.22	1.18
**Total *trans***		**4.53**	**5.07**	**5.20**	**5.17**
C18:2c9t11	CLA (C18:2 c9 t11)	0.15	0.17	0.13	0.17
C18:2t10c12	CLA (C18:2 t10 c12)	0.04	0.08	0.02	0.03
**Total CLA**	**Total CLA**	**0.19**	**0.25**	**0.15**	**0.20**
Unknown	Unknown	0.18	0.20	0.13	0.10

^1^ The single fat composite for each item (heart, kidney, tongue, oxtail) included equal g of fat from each of the three respective individual items from each of three suppliers. ^2^ Oxtail fat is an equal composite of external and internal fat (each composited as stated above); both types were present only for this item.

**Table 10:** Mean and standard error of the mean of individual fatty acids and total of saturated fat (SFA), unsaturated fat (MUFA, PUFA), trans fat, and CLA in separable lean tissue ^1^ from raw U.S. beef offal items, as a percentage of total fatty acids (g/100 g fat).

Fatty Acid Name/Group	Heart	Liver	Kidney	Tongue	Tripe	Oxtail	Bone Marrow	Testicles	Blood
Capric acid (C10:0)	0.01 ± 0.01	<0.01 ± <0.01	0	0.08 ± 0.02	0.02 ± <0.01	0.06 ± 0.01	0.01 ± <0.01	0.06 ± 0.01	0
Lauric acid (C12:0)	0	0	0	0.03 ± 0.01	0	0.07 ± 0.01	<0.01 ± <0.01	0.07 ± 0.01	0
Myristic acid (14:0)	0.29 ± 0.12	0.37 ± 0.02	0.38 ± 0.01	2.49 ± 0.07	2.74 ± 0.10	2.40 ± 0.04	3.36 ± 0.21	1.50 ± 0.03	1.33 ± 0.07
Palmitic acid (16:0)	15.84 ± 0.42	11.38 ± 1.25	16.67 ± 0.60	25.81 ± 0.27	26.33 ± 0.21	23.65 ± 0.51	26.12 ± 0.73	33.79 ± 0.16	15.57 ± 0.22
Margaric acid (17:0)	0.91 ± 0.05	1.06 ± 0.01	0.57 ± 0.01	0.98 ± 0.02	2.30 ± 0.04	1.17 ± 0.03	0.63 ± 0.08	0.64 ± 0.06	0.31 ± 0.01
Stearic acid (18:0)	19.62 ± 0.67	36.61 ± 0.66	16.83 ± 0.37	13.25 ± 0.09	21.8 ± 0.02	15.09 ± 0.03	13.57 ± 0.78	11.33 ± 0.13	21.97 ± 1.60
Arachidic acid (20:0)	0.20 ± 0.01	0.06 ± 0.04	0.39 ± 0.01	0.47 ± 0.11	0.21 ± 0.01	0.14 ± 0.02	0.15 ± 0.04	0.16 ± 0.02	0.10 ± 0.01
Lignoceric acid (24:0)	0.08 ± 0.07	0.22 ± 0.01	0.14 ± 0.01	0.02 ± 0.00	0	0.21 ± 0.03	0	0	0
**Total SFA**	**36.95**	**49.71**	**34.98**	**43.07**	**53.37**	**42.79**	**43.85**	**47.55**	**39.28**
Lauroleic acid (12:1 n3)	<0.01 ± <0.01	0	0	0.03 ± 0.01	0.01 ± 0.01	0.06 ± 0.01	0	0	0
C14:1	0.11 ± 0.05	0.13 ± 0.02	0.23 ± 0.01	1.09 ± 0.02	0.44 ± 0.01	0.46 ± 0.03	0.30 ± 0.02	0.45 ± 0.02	0.17 ± 0.02
Palmitoleic acid (16:1 n7)	0.49 ± 0.05	0.26 ± 0.07	0.16 ± 0.02	0.11 ± 0.01	0.05 ± 0.01	3.10 ± 0.13	1.69 ± 0.04	1.78 ± 0.01	1.43 ± 0.07
C17:1	0.04 ± 0.03	<0.01 ± <0.01	0.15 ± 0.02	0.40 ± 0.03	0.11 ± 0.01	0.98 ± 0.03	0.72 ± 0.04	0.09 ± <0.01	0.22 ± 0.01
Oleic acid (C18:1 c9)	16.21 ± 0.78	14.74 ± 0.14	19.83 ± 2.07	38.70 ± 0.50	37.86 ± 0.14	38.26 ± 0.58	33.24 ± 0.12	20.86 ± 0.28	29.19 ± 0.48
C18:1c11	2.16 ± 0.06	1.92 ± 0.04	3.42 ± 0.19	2.89 ± 0.03	1.72 ± 0.01	2.03 ± 0.04	2.28 ± 0.28	2.63 ± 0.05	1.43 ± 0.03
Eicosenoic acid (C20:1)	0.27 ± 0.03	0.17 ± 0.04	0.34 ± 0.02	0.24 ± <0.01	0.01 ± <0.01	<0.01 ± <0.01	0.17 ± 0.01	0.15 ± 0.01	0
**Total MUFA**	**19.29**	**17.23**	**24.13**	**43.46**	**40.14**	**44.9**	**38.4**	**25.96**	**32.44**
Linolelaidic acid (C18:2)	26.83 ± 2.22	17.26 ± 0.39	23.10 ± 0.84	7.90 ± 0.06	4.61 ± 0.02	6.03 ± 0.19	12.76 ± 0.08	7.20 ± 0.10	19.85 ± 0.98
Linolenic acid (C18:3)	0.61 ± 0.03	1.24 ± 0.07	0.71 ± 0.07	0.39 ± 0.02	0.23 ± 0.02	0.12 ± 0.03	0.58 ± 0.02	0.45 ± 0.03	2.47 ± 0.07
C20:2	0.12 ± 0.03	0.32 ± 0.03	0.38 ± 0.02	0.19 ± 0.10	0.12 ± 0.01	0.01 ± 0.01	0.60 ± 0.25	0.54 ± 0.04	0.31 ± 0.03
Arachidonic acid (C20:4)	13.66 ± 0.37	10.91 ± 0.20	14.13 ± 0.50	2.37 ± 0.05	0.23 ± <0.01	1.40 ± 0.02	2.01 ± 0.08	14.41 ± 0.13	2.48 ± 0.14
C20:5	0.37 ± 0.04	0.41 ± 0.04	0.43 ± 0.03	0.12 ± 0.02	0	0.05 ± <0.01	0.28 ± 0.02	1.06 ± 0.02	0.54 ± 0.03
C22:6	0.13 ± 0.04	0.16 ± 0.04	0.32 ± 0.03	0.10 ± 0.00	0	0.01 ± 0.01	<0.01 ± <0.01	1.52 ± 0.08	0
**Total PUFA**	**41.72**	**30.3**	**39.07**	**11.07**	**5.19**	**7.62**	**16.24**	**25.18**	**25.65**
C18:1t6-8	0.16 ± 0.02	0.12 ± 0.01	0.13 ± 0.01	0.18 ± 0.02	0.19 ± 0.01	0.38 ± 0.03	0.12 ± 0.01	0.16 ± 0.01	0.18 ± 0.03
Elaidic acid (C18:1 t9)	0.13 ± 0.02	0.11 ± 0.02	0.17 ± 0.02	0.28 ± 0.02	0.26 ± 0.02	0.42 ± 0.04	0.11 ± <0.01	0.07 ± 0.01	0.07 ± 0.01
C18:1t10	0.17 ± 0.04	0.17 ± 0.02	0.10 ± 0.01	0.24 ± 0.01	0.14 ± <0.01	2.73 ± 0.06	0.15 ± 0.01	0.17 ± 0.02	0.30 ± 0.03
Vaccenic acid (C18:1 t11)	0.92 ± 0.11	1.59 ± 0.04	0.67 ± 0.09	0.75 ± 0.10	0.20 ± 0.03	0.73 ± 0.06	0.58 ± 0.05	0.43 ± 0.02	0.89 ± 0.06
**Total *trans***	**1.38**	**1.99**	**1.07**	**1.45**	**0.79**	**4.26**	**0.96**	**0.83**	**1.44**
C18:2 c9 t11	0.44 ± 0.08	0.55 ± 0.01	0.40 ± 0.05	0.34 ± 0.06	0.23 ± <0.01	0.30 ± 0.03	0.29 ± 0.01	0.27 ± 0.13	0.86 ± 0.06
C18:2 t10 c12	0.08 ± 0.03	0.04 ± 0.03	0.16 ± 0.01	0.30 ± 0.01	0.02 ± <0.01	0.03 ± 0.01	0.10 ± 0.01	0	0.04 ± 0.01
**Total CLA**	**0.52**	**0.59**	**0.56**	**0.64**	**0.25**	**0.33**	**0.39**	**0.27**	**0.9**
Unknown	0.16 ± 0.02	0.19 ± <0.01	0.17 ± 0.03	0.25 ± 0.05	0.17 ± 0.02	0.10 ± 0.01	0.18 ± 0.04	0.21 ± 0.01	0.29 ± 0.01

^1^ Separable lean tissue includes all lean muscle and organ tissue.

**Table 11:** Percentage of the RDI ^1^ contributed by 100 g of separable lean tissue from raw U.S. beef offal items qualifying for USDA “Excellent Source of” and “Good Source of” extra labeling claims ^2^.

Nutrient	Heart	Liver	Kidney	Tongue	Oxtail ^3^	Tripe ^3^	Bone Marrow ^3^	Testicles ^3^	Blood ^3^
Protein	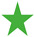	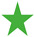	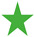	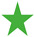	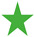	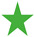		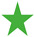	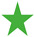
Vitamin A (Retinol)	**X**	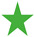	**X**	**X**					
Riboflavin (B_2_)	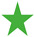	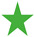	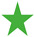	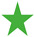					
Pantothenic Acid	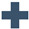	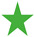	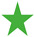	**X**					
Vitamin B_6_	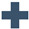	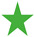	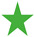	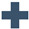					
Niacin (B_3_)	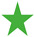	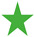	**X**	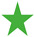					
Vitamin B_12_	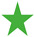	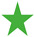	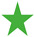	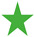					
Vitamin K_1_ (phylloquinone)	**X**	**X**	**X**	**X**	**X**	**X**	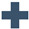	**X**	**X**
Calcium	**X**	**X**	**X**	**X**	**X**	**X**	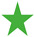	**X**	**X**
Copper	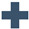	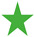	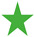	**X**	**X**	**X**	**X**	**X**	**X**
Iron	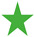	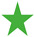	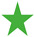	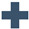	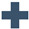	**X**	**X**	**X**	**X**
Manganese	**X**	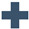	**X**	**X**	**X**	**X**	**X**	**X**	**X**
Phosphorus	**X**	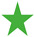	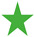	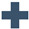	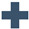	**X**	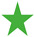	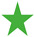	**X**
Zinc	**X**	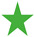	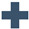	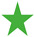	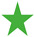	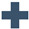	**X**	**X**	**X**

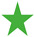
 = Meets “Excellent Source of” certification; 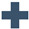 = Meets “Good Source of” certification; **X** = Does not meet either certification. ^1^ Reference daily intakes (RDI) dietary allowance (RDA) is the daily intake level of a nutrient that is considered to be sufficient to meet the requirements of 97–98% of healthy individuals in the United States. ^2^ Providing over 20% of the RDI qualifies the item to be labeled as an “excellent source” of the nutrient. Providing between 10–19% of the RDI qualifies the item to be labeled as a “good source” of the nutrient. ^3^ Oxtail, tripe, bone marrow, testicles, and blood were not analyzed for niacin, riboflavin, pantothenic acid, vitamin B_6_, or vitamin B_12_.

## Data Availability

The original contributions presented in the study are included in the article, further inquiries can be directed to the corresponding author.
